# Conditional Agglomeration in China’s Northeast Rust Belt: Density, Structural Orientation, and Ownership-Mixing Entropy

**DOI:** 10.3390/e28040471

**Published:** 2026-04-20

**Authors:** Omar Abu Risha, Jifan Ren, Mohammed Ismail Alhussam, Mohamad Ali Alhussam

**Affiliations:** 1 School of Economics and Management, Harbin Institute of Technology (Shenzhen), Shenzhen 518055, China; 2 Ningbo China Institute for Supply Chain Innovation-MIT Global Scale Network, Ningbo 315832, China; 3 KIIT School of Management, Kalinga Institute of Industrial Technology, Bhubaneswar 751024, Odisha, India

**Keywords:** labor productivity, agglomeration economies, population density, conditional agglomeration, sectoral entropy, ownership entropy, Northeast China

## Abstract

Northeast China’s rust-belt cities have faced persistent concerns about stagnating labor productivity amid structural change. This paper examines how the productivity payoff to urban density depends on local economic structure and ownership composition using an annual panel of prefecture-level cities. We estimate two-way fixed-effects models with city and year effects and city-clustered standard errors, complemented by dynamic specifications and additional robustness checks. The results show a robust positive within-city association between population density and labor productivity. This density premium is structure-conditioned: the productivity payoff to density is significantly larger in city-years that are more industry-oriented. Information-theoretic measures further show that sectoral and ownership composition matter in distinct ways. A normalized entropy measure based on 19 all-city sectoral employment categories is positively associated with labor productivity, while its interaction with density is negative and significant, indicating that the density premium is weaker in more sectorally balanced city-years. A normalized four-category ownership entropy measure, constructed from SOE, private/self-employed, collective, and other employment shares, is positively associated with labor productivity and interacts positively with density, indicating a stronger density–productivity association in city-years with a more balanced ownership composition. Collectively, the findings suggest that urban density is not a uniform engine of productivity: its payoff depends on whether dense city economies are organized around productive sectoral linkages and a sufficiently balanced ownership environment. Overall, the evidence supports a conditional agglomeration view in which productivity dynamics in Northeast China reflect the interaction of density, structural orientation, sectoral dispersion, and ownership mixing.

## 1. Introduction

Urban agglomeration, the spatial concentration of people and firms, has long been linked to productivity differences across places. Classic microfoundations emphasize that dense environments can raise productivity through sharing (common inputs and infrastructure), matching (thicker labor and supplier markets), and learning (knowledge diffusion and faster imitation) [1]. Empirically, many studies document a positive association between density and output per worker, the so-called “density premium” [2]. At the same time, the literature stresses that interpreting density–productivity relationships requires care because sorting, omitted variables, and reverse causality can bias estimates [3]. Related work also highlights that observed spatial productivity advantages reflect a combination of agglomeration forces and selection mechanisms that may vary across contexts and sectors [4].

A growing concern is that the density premium is not uniform. The effectiveness of density in generating productivity depends on local economic structure and institutional conditions that govern whether proximity translates into specialization, reallocation, and learning. This heterogeneity is particularly salient in rust-belt and shrinking-city settings, where industrial legacies, restructuring pressures, and population outflows may weaken the channels that typically underpin agglomeration economies [5]. These issues are central to Northeast China, Liaoning, Jilin, and Heilongjiang, an old industrial base that has experienced long-run restructuring and renewed policy attention [6]. Recent evidence documents pervasive shrinkage dynamics across the three provinces and emphasizes that decline has become a notable regional phenomenon rather than a set of isolated cases [7,8]. Work on regional decline and structural change in Northeast China further suggests that deindustrialization and weak reallocation capacity can shape growth trajectories during periods of deterioration [9]. Yet, despite the large agglomeration literature, relatively little is known about when density continues to deliver productivity gains in an emerging-economy rust-belt context and which structural features systematically condition those gains.

A second gap concerns how “structure” is conceptualized and measured in density–productivity studies. Existing work often conflates two distinct dimensions: (i) structural orientation (whether a city economy is more industry-oriented versus more service-oriented) and (ii) structural mixing/imbalance (how far a city is from a balanced sectoral distribution, regardless of which sector dominates). Treating these dimensions as interchangeable can obscure what type of structural change matters for agglomeration payoffs [10,11]. To separate them, we complement a directional industry–service orientation metric with information-theoretic entropy measures that quantify distributional mixing independently of direction [12,13,14,15]. We extend the same logic to institutions by constructing ownership-mixing entropy based on employment shares across ownership forms, providing an interpretable descriptor of ownership complexity within cities over time.

This paper addresses these gaps by studying the density–productivity relationship in Northeast China using an annual panel of 34 prefecture-level cities (2007–2021). We estimate two-way fixed-effects models with city and year effects and standard errors clustered at the city level, interpreting coefficients as within-city conditional associations. We then complement static specifications with dynamic fixed-effects models including lagged productivity to account for persistence, treating these dynamic estimates as robustness given the well-known finite-T bias in dynamic fixed-effects settings [16]. Our empirical design allows the density premium to vary with (a) industry–services orientation, (b) sectoral imbalance/mixing captured by entropy measures, and (c) ownership composition and ownership-mixing entropy.

By conditional agglomeration, we mean that the productivity payoff to density is not constant across city-years but varies systematically with local structural and institutional conditions. In this paper, these conditions are captured by structural orientation, sectoral dispersion, and ownership composition. The concept therefore links standard agglomeration mechanisms to the idea that dense urban environments do not generate uniform productivity gains under all configurations of sectoral and organizational structure. The study makes three contributions. First, it provides evidence that the density–productivity relationship in Northeast China is structure-conditioned, clarifying a conceptual distinction between directional structural orientation and undirected mixing/imbalance. Second, it evaluates whether information-theoretic measures of sectoral dispersion and ownership mixing add explanatory power beyond directional structure, helping to identify when such measures are substantively informative for productivity dynamics. Third, the paper documents the role of ownership complexity by examining whether employment-based ownership-mixing entropy is systematically associated with labor productivity and whether such associations differ across provinces, highlighting the ownership-structure dimension of restructuring in China’s Northeast.

The remainder of the paper is organized as follows. Section 2 reviews the related literature and provides the motivation behind our hypotheses. Section 3 describes the data, variable construction, and empirical strategy, including the entropy measures. Section 4 reports the main results and robustness checks. Section 5 discusses implications, limitations, and directions for future research.

## 2. Literature Review

### 2.1. Structural Transformation, Service Quality, and Conditional Agglomeration

The relationship between sectoral composition and labor productivity cannot be reduced to the simple claim that “more services automatically raise productivity.” Structural transformation affects aggregate productivity through a composition effect: productivity rises when labor reallocates toward higher-productivity activities and may fall when labor shifts toward lower-productivity activities [17].

A key implication is that both the direction of structural change, whether a city becomes more industry-oriented or more service-oriented, and the quality of the service sector matter. Producer, or knowledge-intensive, services such as finance, R&D, logistics, and business services often function as intermediate inputs and can strengthen specialization and spillovers, whereas expansion concentrated in lower-productivity local consumer services is less likely to generate comparable productivity externalities. Evidence for China is consistent with this distinction: producer service agglomeration is associated with higher urban productivity, and the strength of this relationship varies across cities [18]. Related work also documents plausible mechanisms through which producer service agglomeration can affect manufacturing outcomes, including scale effects, technology spillovers, and competition [19].

This distinction is particularly relevant in Northeast China, where recent city-level evidence describes a pattern of “premature deindustrialization,” marked by a co-decline in industry and construction employment and linked to weaker regional income trajectories [9]. More broadly, Northeast China is widely described as China’s major old industrial base, shaped by heavy-industry concentration, a strong legacy of planned economy, and path-dependent adjustment pressures [6,9]. In such settings, productivity performance is not determined by a simple “industry versus services” dichotomy but by whether industrial ecosystems remain sufficiently dense while complementary services co-develop in ways that support production, coordination, and upgrading. Consistent with this view, evidence from Chinese cities suggests that coordinated agglomeration of producer services and manufacturing is associated with higher urban green total factor productivity, especially when producer services are more knowledge-intensive [20].

Northeast China may therefore not enjoy automatic agglomeration gains. Comparative city-level evidence finds that enterprise agglomeration is negatively associated with urban productivity in the Northeast, in contrast to other regions, implying that any “density premium” is plausibly contingent on structural conditions [21]. Consistent with this caution, policy evidence from the 2014 revitalization round suggests that industrial-structure transformation and specialization clustering can occur without a statistically significant improvement in technical efficiency, reinforcing the view that restructuring does not necessarily translate into productivity gains [22]. The idea of conditional agglomeration follows directly from standard urban microfoundations. Density may improve productivity through sharing, matching, and learning, but the strength of these channels depends on how the local economy is organized. Where dense cities are anchored in stronger input–output linkages, thicker labor-market matching, and better opportunities for knowledge circulation, the productivity return to density should be larger. Where these structural conditions are weaker, the same increase in density may yield a smaller payoff. Agglomeration is therefore not a uniform treatment, but a relationship whose strength depends on sectoral and institutional context.

Because theory implies competing mechanisms, we do not impose a single sign a priori. Instead, we test whether the density premium is conditioned by both structural direction and sectoral dispersion/balance.

**H1:**  *Structural characteristics condition the density premium. In particular, both sectoral orientation and sectoral dispersion, captured by entropy-based measures, may moderate the density–productivity relationship in restructuring regions.*

### 2.2. Ownership Structure, Financing Frictions, and Density Premium

A central mechanism is differential access to finance. Using firm-level evidence on credit rationing, Gao et al. [23] find that state ownership is associated with a lower likelihood of credit rationing in China, i.e., SOEs are less likely to be credit-rationed, highlighting how ownership can systematically affect financing constraints and investment capacity [23]. At the same time, private firms commonly face ownership-based frictions in formal finance. Bai et al. [24] show that private firms are significantly less funded through formal channels such as bank loans than state-owned firms and therefore rely more on alternative finance such as trade credit; this provides direct evidence of “ownership discrimination” in financing and a clear substitution toward non-bank finance. This pattern is consistent with earlier evidence of bank discrimination against private firms in China’s transition context [25].

These ownership-linked financing asymmetries imply systematic differences in sectoral positioning and firm behavior, which matter for agglomeration. Consistent with evidence that changes in the size of the SOE sector affect entrepreneurship and firm formation, SOE downsizing in the late 1990s is associated with improvements in the quantity and quality of entrepreneurship [26]. SOEs are more prevalent in capital-intensive and policy-relevant activities, while private firms are more represented in competitive segments, often under tighter financial constraints, shaping the extent to which dense urban environments translate into upgrading, coordination, and spillovers. In this sense, ownership composition can moderate the density premium through at least two mechanisms. First, in SOE-intensive cities, implicit guarantees and soft budget constraints may relax financing constraints and sustain higher leverage but may also distort credit allocation and promote inefficiency, making the net productivity payoff from density theoretically ambiguous [27]. Second, in private, intensive cities, the density premium may be weaker if private activity is disproportionately concentrated in fragmented low-value segments and if financing frictions constrain longer-horizon investments that support learning and spillovers [24,25]. We therefore measure ownership structure using both SOE/private employment shares and ownership entropy [12] to test whether dispersion adds information beyond dominance.

**H2:**  *The density–productivity association is conditioned by ownership structure: it varies with SOE and private employment shares and is further moderated by ownership-share dispersion (ownership entropy), over and above the effects of the shares themselves.*

## 3. Data, Variables, and Empirical Strategy

### 3.1. Study Area, Sample, and Panel Structure

Using city-level data from the China City Statistical Yearbook and provincial yearbooks for Liaoning, Jilin, and Heilongjiang, this study examines whether the density–productivity association varies with sectoral structure and ownership composition. The sample period (2007–2021) is chosen to cover a sustained phase of restructuring in Northeast China and to ensure consistent data availability across cities and variables. The study variables are listed in Table 1.

### 3.2. Empirical Models

#### 3.2.1. Fixed-Effects Framework and Interaction Design

The empirical strategy proceeds in three steps. First, a dynamic two-way fixed-effects model is estimated to account for persistence in productivity. Second, the density premium is allowed to vary with structural direction by including a density–structure interaction term. Third, information-theoretic measures are introduced as alternative moderators to test whether *mixing/imbalance* (sectoral and ownership dispersion) provides explanatory power beyond directional indicators

(1) $${LP}_{it}=\rho \;{LP}_{i,t-1}+\beta_{1}\;{Dens}_{i,t}+\beta_{2}\;{Struct}_{it}+\beta_{3}\;{Soe\;\_Emp}_{it}\;\;\;\;\;\;\;\;\;\;\;\;\;\;\;\;\;\;\;\;\;\;\;\;\;\;\;\;\;\;\;\;\;\;\;\;\;\;\;\;\;\;\;\;\;\;+\beta_{4}\;{Pri\;\_Emp}_{it}+\gamma_{1}\;{Edu}_{it}+\gamma_{2}\;{Health}_{it}+\mu_{i}+\lambda_{t}+\varepsilon_{it}$$

To test whether the density premium varies with structural orientation:

(2) $${LP}_{it}\;=\;\rho \;{LP}_{i,t-1}\;+\;\beta_{1}\;{Dens}_{i,t}\;+\;\beta_{2}\;{Struct}_{it}\;+\;\beta_{3}\;\left({Dens}_{i,t}\;\times \;{Struct}_{it}\right)\;\;\;\;\;\;+\;\beta_{4}\;{Soe\;\_Emp}_{it}\;+\;\beta_{5}\;{Pri\;\_Emp}_{it}\;+\;\;\gamma_{1}\;{Edu}_{it}\;+\;\gamma_{2}\;{Health}_{it}\;+\;\mu_{i}\;+\;\lambda_{t}\;+\;\varepsilon_{it}\;$$

where $\left(\mu_{i}\right)$ is the city fixed effects and $\left(\lambda_{t}\right)$ is the year fixed effects. A practical concern in dynamic fixed-effects models is the finite-T bias. Intuitively, once city fixed effects are removed, the lagged dependent variable remains mechanically correlated with the transformed error term when the time dimension is limited. As a result, the coefficient on lagged productivity, and potentially the coefficients on correlated regressors, may be biased in conventional dynamic FE estimation. This bias becomes smaller as the number of time periods increases. For this reason, we treat the conventional dynamic FE estimates as supportive rather than decisive and complement them with additional dynamic-panel checks. We also assessed potential multicollinearity using variance inflation factors (VIFs) from auxiliary OLS specifications with the same substantive regressors and year effects; the results, reported in Appendix A Table A5, do not indicate a level of collinearity severe enough to affect the qualitative interpretation of the main coefficients.

#### 3.2.2. Sectoral and Ownership Entropy Measures

To capture how sectoral dispersion and ownership mixing may condition the density–productivity association, we construct two entropy-based sets of moderators. In the main analysis, we use normalized entropy measures so that the indicators are bounded and comparable in interpretation across observations. All entropy measures are computed from observed city-year employment shares and are included only when the relevant component shares are available and the corresponding total employment mass is positive.

i. Sectoral entropy based on 19 all-city employment categories

The main sectoral entropy measure is computed from employment shares across 19 all-city sectoral categories reported in the city yearbooks. Let $E_{kit}$ denote employment in sector k  for city i  in year t, where k=1,…,19, and define total sectoral employment as

(3) $$S_{\left(it\right)}^{\left(sector\right)}=\sum {\left(k=1\right)}^{19}E_{\left(kit\right)}$$

The corresponding sectoral employment share is

(4) $$p_{kit}=\frac{E_{kit}}{S_{it}^{sector}}$$

Sectoral Shannon entropy is then defined as

(5) $$H_{it}^{sector19}=-\sum_{k=1}^{19}p_{kit}ln(p_{kit})$$

and the normalized sectoral entropy used in the main regressions is

(6) $${\tilde{H}}_{it}^{sector19}=\frac{H_{it}^{sector19}}{ln(19)}$$

Higher values indicate a more evenly distributed all-city employment structure across sectors, whereas lower values indicate stronger concentration in a smaller set of sectors.

ii. Ownership entropy based on four ownership categories

The main ownership entropy measure is constructed from city-year employment shares in four ownership groups: SOEs, private/self-employed units, collective units, and other ownership types. Let the total ownership mass be

(7) $$S_{it}^{own4}=Emp_{SOE,it}+Emp_{PriSelf,it}+Emp_{Collective,it}+Emp_{Other,it}$$

The corresponding ownership shares are

(8) $$q_{it}^{SOE}=\frac{Emp_{SOE,it}}{S_{it}^{own4}},q_{it}^{PriSelf}=\frac{Emp_{PriSelf,it}}{S_{it}^{own4}},q_{it}^{Collective}=\frac{Emp_{Collective,it}}{S_{it}^{own4}},q_{it}^{Other}=\frac{Emp_{Other,it}}{S_{it}^{own4}}$$

The four-category ownership entropy is

(9) $$H_{it}^{own4}=-\left(q_{it}^{SOE}lnq_{it}^{SOE}+q_{it}^{PriSelf}lnq_{it}^{PriSelf}+q_{it}^{Collective}lnq_{it}^{Collective}+q_{it}^{Other}lnq_{it}^{Other}\right)$$

and the normalized form used in the main analysis is

(10) $${\tilde{H}}_{it}^{own4}=\frac{H_{it}^{own4}}{ln(4)}$$

Higher values indicate a more balanced local ownership composition across the four ownership groups.

iii. Binary ownership entropy: SOE versus private/self-employed

As a simpler alternative ownership measure, we also construct a binary entropy index based only on SOE and private/self-employed employment. Define the binary ownership mass as

(11) $$S_{it}^{own2}=Emp_{SOE,it}+Emp_{PriSelf,it}$$

with normalized shares

(12) $$q_{it}^{SOE,2}=\frac{Emp_{SOE,it}}{S_{it}^{own2}},q_{it}^{PriSelf,2}=\frac{Emp_{PriSelf,it}}{S_{it}^{own2}}$$

The binary ownership entropy is

(13) $$H_{it}^{own2}=-\left(q_{it}^{SOE,2}lnq_{it}^{SOE,2}+q_{it}^{PriSelf,2}lnq_{it}^{PriSelf,2}\right)$$

and the normalized form is

(14) $${\tilde{H}}_{it}^{own2}=\frac{H_{it}^{own2}}{ln(2)}.$$

This binary measure provides a parsimonious indicator of balance between state and private/self-employed employment.

#### 3.2.3. Dynamic Fixed-Effects Specifications with Entropy Moderators

The entropy measures enter the empirical analysis as moderators of the density–productivity association. Let $LP_{it}$ denote log labor productivity, $Dens_{it}$ denote log population density, and $Struct_{it}$ denote the log industry-to-services ratio. The sectoral entropy specification is written as

(15) $$LP_{it}=\rho LP_{i,t-1}+\beta_{1}Dens_{it}+\beta_{2}{\tilde{H}}_{it}^{sector19}+\beta_{3}\left(Dens_{it}\times {\tilde{H}}_{it}^{sector19}\right)+\beta_{4}SOE_{Emp_{it}}\;\;\;\;+\;\beta_{5}Pri\_Emp_{it}+\gamma_{1}Edu_{it}+\gamma_{2}Health_{it}+\mu_{i}+\lambda_{t}+\varepsilon_{it}.$$

The four-category ownership entropy specification is

(16) $$LP_{it}=\rho LP_{i,t-1}+\beta_{1}Dens_{it}+\beta_{2}{\tilde{H}}_{it}^{own4}+\beta_{3}\left(Dens_{it}\times {\tilde{H}}_{it}^{own4}\right)+\beta_{4}Struct_{it}\;\;+\;\gamma_{1}Edu_{it}+\gamma_{2}Health_{it}+\mu_{i}+\lambda_{t}+\varepsilon_{it}.\;$$

The binary ownership entropy specification is

(17) $$LP_{it}=\rho LP_{i,t-1}+\beta_{1}Dens_{it}+\beta_{2}{\tilde{H}}_{it}^{own2}+\beta_{3}\left(Dens_{it}\times {\tilde{H}}_{it}^{own2}\right)+\beta_{4}Struct_{it}\;\;+\;\gamma_{1}Edu_{it}+\gamma_{2}Health_{it}+\mu_{i}+\lambda_{t}+\varepsilon_{it}.\;$$

These specifications allow us to distinguish among three related but conceptually different dimensions of restructuring. Structural direction is captured by $Struct_{it}$, that is, the relative orientation toward industry versus services. Sectoral dispersion is captured by normalized entropy across 19 all-city sectoral employment categories. Ownership mixing is captured by normalized entropy across either four ownership groups or, in the simpler specification, SOE versus private/self-employed employment. This setup allows a direct empirical test of whether the density premium varies primarily with economic orientation, with the breadth of sectoral distribution, or with the balance of local ownership composition.

## 4. Results

### 4.1. Baseline Density Premium and Structural Conditioning

The results proceed in three steps. Table 2 reports the baseline within-city density–productivity association using two-way fixed effects with city and year effects, and city-clustered standard errors, and tests structural conditioning through the interaction between density and the industry-to-services ratio. Table 3 then examines whether the density premium varies with the ownership environment through the interaction between density, and mean-centered SOE and private employment shares. Table 4 extends the analysis by introducing refined information-theoretic measures, namely normalized sectoral entropy based on 19 all-city sectoral employment categories, a normalized four-category ownership entropy measure, and a simpler binary SOE–private/self-employed ownership entropy measure.

**Figure 1:**
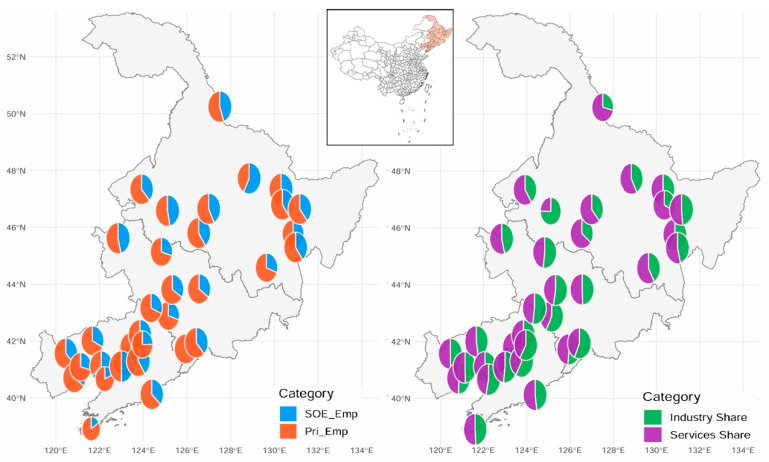
Spatial distribution of ownership and sectoral composition across sample cities.

To provide intuition for the structural and ownership variables used in the regressions, Figure 1 maps the underlying sectoral and ownership shares across sample cities. Panel (a) shows SOE versus private employment shares, and Panel (b) shows industry versus services shares. The figure is descriptive, but it shows that composition is not randomly distributed across space. In Panel (a), many southern and coastal cities display visibly larger private employment shares, whereas several inland and northern cities show a more balanced ownership mix or relatively larger SOE shares. In Panel (b), services account for the larger share in most cities, but the degree of service dominance varies clearly across locations. Taken together, the maps indicate substantial spatial heterogeneity in both ownership and sectoral composition across Northeast China.

Table 2 reports two-way fixed-effects estimates relating population density to labor productivity in Northeast Chinese cities. In the baseline static FE model, ln(Dens) is positive and statistically significant $\left(0.904,\;s.e.\;0.301\right)$, indicating a positive within-city association between density and productivity over time after controlling for covariates and common year shocks. The structural orientation measure ln(Struct) is also positive and significant $\left(0.271,\;s.e.\;0.058\right)$, where ln(Struct)=ln(industry/service) and higher values indicate a more industry-oriented structure.

Because density, structural orientation, and ownership composition may be jointly determined with labor productivity in a short dynamic panel, the baseline FE estimates are complemented by two additional robustness checks for the core interaction model, reported in Appendix A Table A4. A two-step robust System-GMM specification preserves the positive interaction between density and structural orientation, and an Anderson–Hsiao IV specification in first differences yields the same qualitative conclusion. These additional estimates strengthen confidence in the interaction pattern while still being interpreted as conditional associations rather than fully identified causal effects.

In dynamic specifications that include lagged productivity, productivity is highly persistent (0.469–0.453, *p* < 0.01) and the density premium remains positive and significant (0.946–1.078, *p* < 0.01); the density × structure interaction also remains positive though attenuated (0.099, s.e. 0.038).

### 4.2. Ownership Environment as a Moderator of the Density Premium

**Figure 2:**
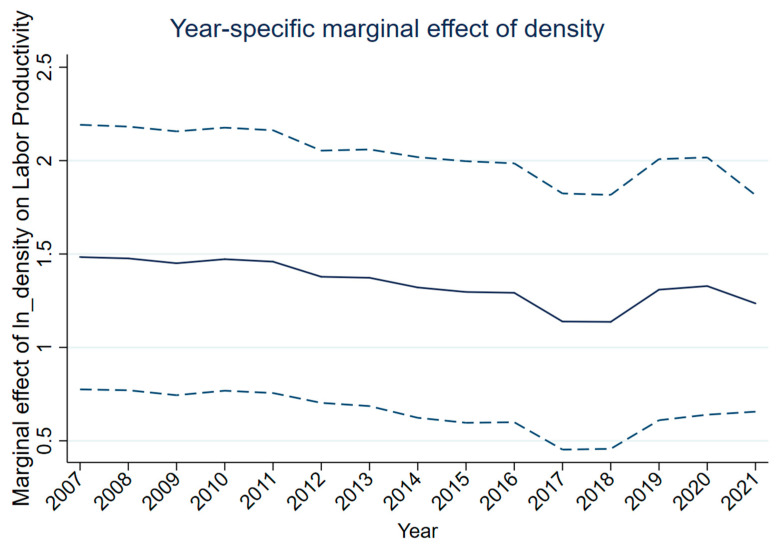
Marginal effect of population density on labor productivity. The solid line shows the estimated marginal effect. The dashed lines denote 95% confidence intervals.

This study next examines whether the density–productivity association varies systematically with ownership intensity (Table 3). Interacting density with the mean-centered private employment share yields a negative and precisely estimated interaction term (ln(Dens) × private share: −0.944, s.e. 0.185, *p* < 0.01), indicating that the density premium is attenuated in more private-intensive city-years, relative to the sample mean. This pattern should be interpreted cautiously because the private-share measure is employment-based and includes private/self-employed activity, so higher private intensity in this sample need not correspond to the type of formal, high-productivity private-sector upgrading often emphasized in the broader Chinese context. By contrast, the corresponding interaction with the mean-centered SOE employment share is positive but not statistically distinguishable from zero (0.175, s.e. 0.187). Figure 2 visualizes the implied marginal effect of density across low, mean, and high values of private-sector intensity.

Figure 2 shows the estimated year-by-year marginal effects of ln(Dens) on ln(LP) from a fixed-effects model that shows the interactions of ln(Dens) with year indicators (city FE; year FE; baseline controls). Dashed lines denote 95% confidence intervals. The figure is descriptive, documenting temporal heterogeneity in within-city associations and serving as a diagnostic that motivates subsequent tests of structural conditioning.

**Figure 3:**
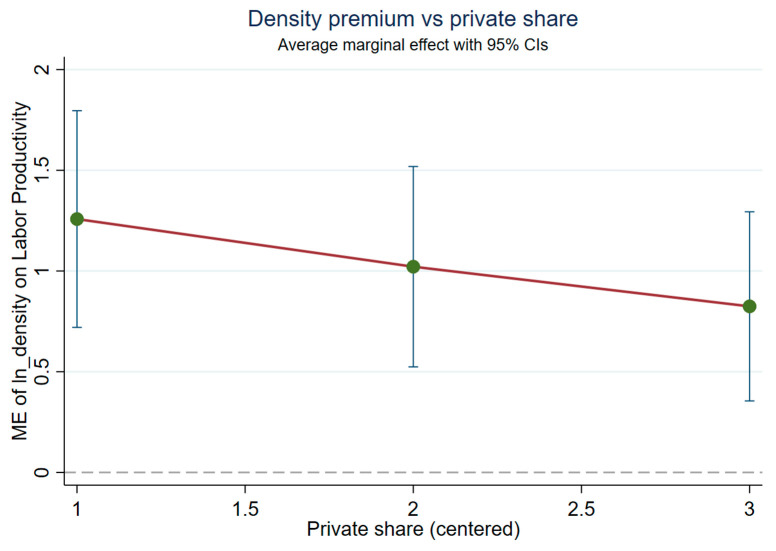
Density premium by private-sector intensity (average marginal effects).

Figure 3 shows a monotonic decline in the estimated density premium as the private employment share rises, consistent with the negative interaction term in the dynamic FE model. The implied density–productivity association remains positive throughout the plotted range but is materially attenuated in more private-intensive city-years.

### 4.3. Entropy Indicators: Sectoral Dispersion and Ownership Mixing

Table 4 reports the dynamic fixed-effects results using refined sectoral and ownership entropy measures. Column 1 introduces a normalized 19-sector entropy measure computed from all-city sectoral employment shares, together with its interaction with population density. The coefficient on H_sector19 is positive and statistically significant (1.653, s.e. 0.343, *p* < 0.01), indicating that greater sectoral diversity is positively associated with labor productivity. At the same time, the interaction between density and sectoral entropy is negative and highly significant (−1.727, s.e. 0.267, *p* < 0.01), suggesting that the density premium is weaker in more sectorally balanced city-years.

Columns 2 and 3 report the ownership–entropy results. Column 2 uses a normalized four-category ownership entropy measure constructed from employment shares in SOEs, private/self-employed units, collective units, and other ownership types. This measure is positive and statistically significant (1.075, s.e. 0.370, *p* < 0.01). Its interaction with density is also positive and significant (0.747, s.e. 0.349, *p* < 0.05), indicating that the density–productivity association is stronger in city-years with a more balanced ownership composition. Column 3 shows that this pattern is also present under the simpler binary SOE–private/self-employed entropy specification, with a positive entropy coefficient (1.195, s.e. 0.231, *p* < 0.01) and a positive density interaction (0.330, s.e. 0.191, *p* < 0.10). Overall, Table 4 shows that both sectoral and ownership entropy contain productivity-relevant information in the panel.

### 4.4. Robustness and Cross-Province Heterogeneity

Robustness checks in Appendix A Table A1 show that the key density × structure interaction is not driven by any single city or sample restriction. In leave-one-city-out exercises, the interaction coefficient remains positive and statistically significant in all 34 replications, varying within a relatively narrow range from 0.0829 to 0.1204. The same qualitative result holds when excluding 2021, removing potentially influential cities (cities 2 and 23), or dropping the major regional core cities. Additionally, Appendix A Table A2 and Table A3 show that both the sectoral entropy and ownership entropy results remain stable across alternative samples and over-control specifications, supporting the robustness of the main entropy-based findings.

**Figure A1:**
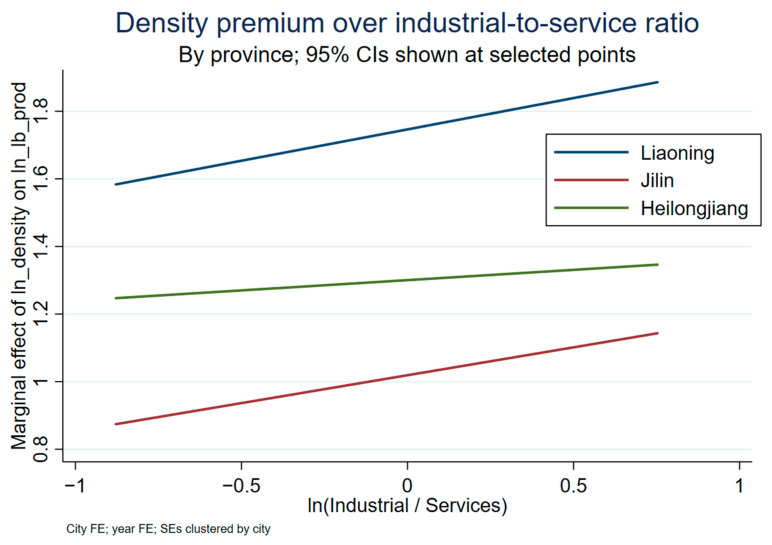
Province-specific density premium over industrial-to-services orientation.

**Figure A2:**
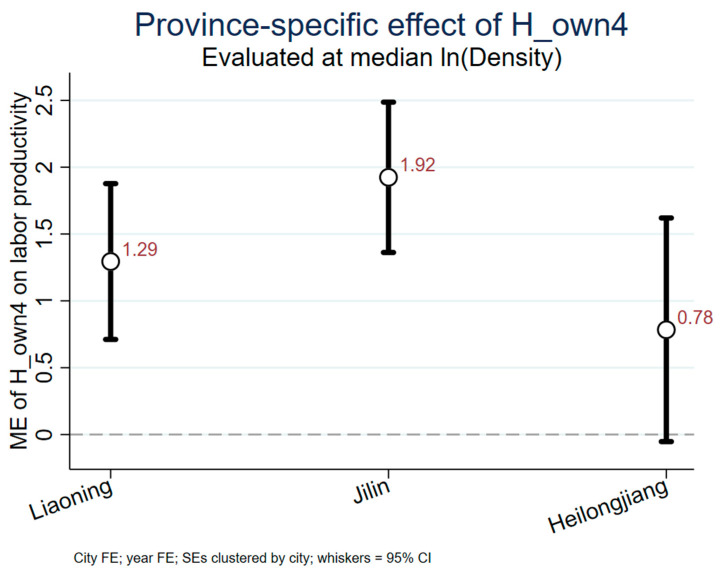
Province-specific association of ownership entropy (H_own4) with labor productivity.

Appendix A Figure A1 and Figure A2 indicate that the provincial heterogeneity is substantively meaningful rather than merely descriptive. Figure A1 shows that the implied density premium rises with structural orientation in all three provinces, but the magnitude differs, with Liaoning exhibiting a consistently larger density premium than Jilin and Heilongjiang across the plotted range. Figure A2 shows that the ownership–entropy association is clearly stronger in Liaoning and Jilin than in Heilongjiang, where the estimated effect is smaller and less precisely estimated. A plausible institutional interpretation is that ownership entropy becomes productivity-relevant only when a more balanced ownership composition is able to support effective coexistence across organizational forms. In Liaoning and Jilin, a more even distribution across SOEs, private/self-employed units, collective units, and other ownership types may better reflect an environment in which different organizational forms are able to interact through competition, supplier linkages, labor reallocation, and complementary roles in local production systems. In other words, ownership entropy appears to be productivity-relevant where it captures productive organizational coexistence, but less so where it reflects balance without effective reallocation. In Heilongjiang, the weaker and less precisely estimated association suggests that ownership entropy by itself may be less able to translate into productivity gains when restructuring constraints remain more binding. This interpretation is consistent with studies of resource-based cities in Northeast China reporting stronger urban transition performance and higher economic resilience in Liaoning and Jilin than in Heilongjiang [28,29,30].

## 5. Discussion and Policy Implications

### 5.1. Structure-Conditioned Agglomeration: Density Premium and Sectoral Orientation, and Entropy-Based Conditioning

The combined evidence supports a conditional agglomeration narrative. A positive density–productivity association is consistent with foundational evidence that productivity rises with the density of economic activity, reflecting localized externalities [2]. Beyond this baseline, the main result is that the density premium is structure-dependent: the significant density–structure interaction indicates that agglomeration gains are larger when city-years are more industry-oriented. This is consistent with micro-foundations emphasizing that agglomeration operates through sharing, matching, and learning and that the strength of these channels depends on the organization of production and the intensity of input–output linkages [1]. It also echoes work separating agglomeration forces from selection mechanisms, implying that observed productivity advantages can reflect a combination of external economies and compositional changes, precisely the type of heterogeneity our interaction design is intended to capture [4]. China-specific evidence at both firm and city levels likewise supports the view that agglomeration effects are substantial and that industrial composition conditions these benefits [31,32,33].

At the same time, the entropy results show that conditional agglomeration in Northeast China is shaped not only by structural direction but also by how sectoral and ownership compositions interact with density. In other words, the productivity return to density varies not only with whether a city-year is more industry- or service-oriented but also with how employment is distributed across sectors and ownership forms.

### 5.2. Refined Sectoral Entropy: Diversity, Balance, and the Density Premium

The revised sectoral entropy results add an important new dimension to the interpretation of conditional agglomeration. Using a normalized entropy measure computed from 19 all-city sectoral employment categories, we find that sectoral entropy is positively associated with labor productivity, indicating that broader sectoral diversity is linked to higher productivity levels within cities over time. At the same time, the interaction between density and sectoral entropy is negative and statistically significant, suggesting that the productivity payoff to density is weaker in more sectorally balanced city-years.

This pattern implies that sectoral diversity and density do not operate in a uniformly reinforcing way. A more diversified city economy may be associated with higher productivity in levels, but the marginal productivity return to density appears stronger when employment is less evenly spread across sectors. In the context of Northeast China, this is consistent with the idea that dense urban environments may yield stronger productivity gains when they remain anchored in relatively concentrated, input-linked, and tradable structures, rather than in broadly dispersed sectoral compositions. Put differently, diversity may support productivity in general, while the density premium itself may be amplified when urban activity is organized around stronger production linkages rather than evenly balanced sectoral breadth.

Accordingly, the evidence indicates that information-theoretic sector measures are substantively informative when constructed using richer sectoral detail. Sectoral entropy should therefore be treated not merely as a diagnostic balance measure, but as an additional channel through which the density–productivity association is conditioned in restructuring cities.

### 5.3. Ownership Structure and Institutional Frictions: Shares, Mixing Entropy, and Productivity

Turning to ownership composition, we find that within-city changes in SOE and private employment shares are associated with labor productivity, but we interpret these coefficients as conditional associations rather than causal effects. In particular, the negative coefficient on private employment share should be interpreted cautiously. In our setting, this variable is employment-based and includes private/self-employed activity, so a higher value does not necessarily indicate the type of formal, high-productivity private-sector dynamism often emphasized in the broader Chinese context. In Northeast China’s restructuring cities, a larger private employment share may instead reflect a composition with greater weight in smaller-scale, lower-capitalized, or self-employment-intensive segments. The estimate is therefore better read as a contextual within-city conditional association than as a general statement about the productivity effects of private-sector development.

This interpretation also helps in reading the negative interaction between density and private employment share reported in Table 3. Rather than implying that private activity is inherently productivity-reducing, the result suggests that the productivity payoff to density is weaker in city-years where private/self-employed employment accounts for a larger share of total employment in this sample. From a misallocation perspective, ownership-linked frictions, especially unequal access to capital and factor-market distortions, can shape productivity outcomes, making share coefficients sensitive to local selection and sectoral composition [34,35].

More importantly, ownership dispersion and mixing, captured by information-theoretic entropy measures, are strongly and robustly associated with labor productivity in our dynamic fixed-effects models. The main ownership measure is a normalized four-category entropy index constructed from employment shares in SOEs, private/self-employed units, collective units, and other ownership types. This measure is positively associated with labor productivity and also interacts positively with density, indicating that the density–productivity association is stronger in city-years with a more balanced ownership composition. A simpler binary SOE–private/self-employed entropy measure yields the same qualitative pattern.

A plausible mechanism is that higher ownership entropy reflects a local environment in which different organizational forms coexist more evenly, rather than one in which a single ownership type dominates local employment. In such settings, ownership balance may be associated with stronger competitive pressure, broader buyer–supplier linkages, and greater scope for labor and resource reallocation across firms and sectors. It may also create more opportunities for organizational complementarity, in the sense that different ownership forms play different roles within the local production system. From this perspective, the positive association between ownership entropy and productivity is consistent with channels involving competition, spillovers, and allocation efficiency.

This result is consistent with evidence that state involvement may be associated with lower productivity and capital misallocation under some institutional settings, while reforms affecting the state–nonstate boundary can be productivity-relevant [35,36].

Complementary city-level evidence also suggests that SOE presence can influence local economic dynamics, including by inhibiting manufacturing employment growth and slowing the expansion of non-SOEs in SOE-intensive cities [37,38]. In this sense, an ownership-mixing entropy measure can proxy for broader institutional and competitive conditions rather than a purely mechanical diversification effect. Accordingly, we treat ownership entropy primarily as an employment-based structural descriptor that summarizes ownership balance and coexistence, rather than as a direct policy lever with a clean causal interpretation [39].

### 5.4. Limitations, Implications, and Future Research

This study has several limitations that should be kept in mind when interpreting the results. First, the empirical design is based on within-city panel variation with fixed effects, so the estimates are best interpreted as conditional relationships rather than fully identified causal effects. Second, the entropy measures are constructed as aggregate city-level descriptors and therefore do not directly identify the firm-level channels through which density, sectoral composition, and ownership balance translate into productivity differences. In particular, the sectoral entropy measure is computed from 19 all-city sectoral employment categories, while the main ownership entropy measure is constructed from four ownership groups, namely SOEs, private/self-employed units, collective units, and other ownership types. These measures capture meaningful structural variation, but they do not by themselves distinguish among underlying mechanisms such as competition, sorting, upgrading, or resource reallocation. Third, the city-year data do not permit a consistent decomposition of private employment into formal private firms versus self-employment, or into higher-skill versus lower-skill private activity, over the full sample period. This limits how precisely the negative private-share coefficient can be interpreted. Fourth, the province-specific heterogeneity in the estimates suggests that the observed patterns should not be interpreted as mechanically uniform across Liaoning, Jilin, and Heilongjiang.

From a policy perspective, the findings suggest that the productivity payoff to density depends on how restructuring is organized. Stronger returns to density appear more likely when urban development remains linked to productive, input-connected activities and when local ownership structures are sufficiently balanced to support coexistence across different organizational forms. More broadly, the results indicate that density, sectoral composition, and ownership balance should be considered jointly rather than in isolation when evaluating restructuring strategies in Northeast China. Future research can build on this framework by linking city-level entropy measures to firm-level reallocation channels, incorporating more direct institutional indicators, and exploiting policy shocks or quasi-experimental variation to sharpen causal interpretation.

## Figures and Tables

**Table 1:** Definitions and measurement of model variables.

Variable (Symbol)	Used in Model as	Definition/Construction	Unit
Labor productivity (LP)	ln(${LP}_{it}$)	$\ln\left(\frac{GDP_{it}}{{\mathrm{UrbanPersonsEmp}}_{it}+{\mathrm{Emp}\_\mathrm{PrivateSelfEmployed}}_{it}}\right)$	log units
Population density (DENS)	ln(${Dens}_{it}$)	$\ln\left(\frac{\mathrm{TotalPo}p_{\mathrm{it}}}{\mathrm{Are}a_{\mathrm{it}}}\right)$	log persons/${km}^{2}$
Industry–service structure	ln(${Struct}_{it}$)	$\ln\left(\frac{\mathrm{industry}\_pc_{\mathrm{it}}}{\mathrm{service}\_pc_{\mathrm{it}}}\right)$	log ratio
SOE_EMP employment share	${Soe\_Emp}_{it}$	$\frac{{\mathrm{SOEworkers}}_{\mathrm{it}}}{{\mathrm{ActiveLaborForce}}_{\mathrm{it}}}$	share (0–1)
Private employment share	${Pri\_Emp}_{it}$	$\frac{{\mathrm{Private}\&\mathrm{SelfEmp}}_{\mathrm{it}}}{{\mathrm{ActiveLaborForce}}_{\mathrm{it}}}$	share (0–1)
Education (preferred)	${\mathrm{Edu}}_{it}$	$100\;\times \;\left(\frac{\mathrm{TertiaryStudent}s_{\mathrm{it}}}{\mathrm{TotalPo}p_{\mathrm{it}}}\right)$	%
Health index	${\mathrm{Health}}_{it}$	$\frac{z\left({\mathrm{Hospitals}}_{it}\right)+z\left({\mathrm{Beds}}_{it}\right)+z\left({\mathrm{Doctors}}_{it}\right)}{3}$	index
Time fixed effects	i.year	Year dummies	
City fixed effects	fe (xtreg, fe)	City fixed effects	

**Table 2:** Two-way fixed-effects estimates: density premium and structural conditioning.

Variables	Static FE Core	Static FE Dens × Struct	Dynamic FE	Dynamic FE + Interaction
ln(Dens)	0.904 ***	1.091 ***	0.946 ***	1.078 ***
	(0.301)	(0.293)	(0.290)	(0.276)
ln(Struct)	0.271 ***	0.242 ***	0.206 ***	0.189 ***
	(0.0579)	(0.0528)	(0.0360)	(0.0325)
ln(Dens) × ln(Struct)		0.141 ***		0.0994 **
		(0.0507)		(0.0380)
Soe_Emp	0.942 ***	0.846 ***	0.627 ***	0.569 ***
	(0.204)	(0.188)	(0.144)	(0.134)
Pri_Emp	−0.786 **	−0.908 ***	−0.741 ***	−0.836 ***
	(0.304)	(0.296)	(0.253)	(0.260)
Edu	−0.0371	−0.0309	−0.0317	−0.0251
	(0.0578)	(0.0555)	(0.0398)	(0.0381)
Health	−0.525	−0.473	−0.252	−0.223
	(0.482)	(0.444)	(0.298)	(0.274)
L.ln(LP)			0.469 ***	0.453 ***
			(0.0633)	(0.0634)
Constant	1.686 ***	0.970 ***	0.966 ***	1.732 ***
	(0.287)	(0.323)	(0.312)	(0.300)
Year FE	Yes	Yes	Yes	Yes
City FE	Yes	Yes	Yes	Yes
Observations	485	485	451	451
R^2^	0.774	0.783	0.778	0.784
Number of Cities	34	34	34	34

Notes: Standard errors in parentheses. City and year fixed effects included; year coefficients omitted for brevity. *** *p* < 0.01, ** *p* < 0.05.

**Table 3:** Dynamic fixed-effects models with centered ownership moderators.

Variables	Dens × Private	Dens × SOE
L.ln(LP)	0.391 ***	0.468 ***
	(0.0663)	(0.0629)
ln(Dens)	1.034 ***	0.991 ***
	(0.247)	(0.296)
Pri_Emp (c.)	−0.475 **	−0.708 ***
	(0.203)	(0.258)
ln(Dens)× Pri_Emp	−0.944 ***	
	(0.185)	
Soe_Emp	0.416 ***	0.611 ***
	(0.106)	(0.151)
ln(Dens) × Soe_Emp		0.175
		(0.187)
ln(Struct)	0.232 ***	0.215 ***
	(0.0314)	(0.0384)
Edu	−0.0234	−0.0299
	(0.0376)	(0.0381)
Health	0.106	−0.257
	(0.231)	(0.286)
Constant	0.873 ***	0.762 ***
	(0.202)	(0.214)
City FE	Yes	Yes
Year FE	Yes	Yes
Observations	451	451
R^2^	0.813	0.780
Number of cities	34	34

Notes: Standard errors in parentheses. City and year fixed effects included; year coefficients omitted for brevity. *** *p* < 0.01, ** *p* < 0.05.

**Table 4:** Dynamic fixed-effects models augmented with entropy indicators.

Variable	Dyn FE + H_Sector19	Dyn FE + H_own4	Robust Dyn + H_own2
L.ln(LP)	0.393 ***	0.462 ***	0.397 ***
	(0.0673)	(0.0751)	(0.0740)
ln(Dens)	2.415 ***	0.00595	0.460
	(0.794)	(0.797)	(0.727)
H_sector19 (normalized)	1.653 ***		
	(0.343)		
ln(Dens) × H_sector19	−1.727 ***		
	(0.267)		
H_own4 (4-category, normalized)		1.075 ***	
		(0.370)	
ln(Dens) × H_own4		0.747 **	
		(0.349)	
H_own2 (SOE vs. Private, normalized)			1.195 ***
			(0.231)
ln(Dens) × H_own2			0.330 *
			(0.191)
ln(Struct)		0.254 ***	0.286 ***
		(0.0374)	(0.0400)
Soe_Emp	0.535 ***		
	(0.148)		
Pri_Emp	−1.156 ***		
	(0.226)		
Education	0.0229	−0.0453	−0.0370
	(0.0390)	(0.0595)	(0.0347)
Health	−0.189	0.261	0.172
	(0.288)	(0.337)	(0.295)
Constant	−0.0142	0.144	−0.0832
	(0.458)	(0.388)	(0.385)
City FE	Yes	Yes	Yes
Year FE	Yes	Yes	Yes
Observations	440	442	442
R^2^	0.787	0.777	0.805
Number of cities	34	34	34

Notes: Standard errors in parentheses. City and year fixed effects included; year coefficients omitted for brevity. *** *p* < 0.01, ** *p* < 0.05, * *p* < 0.10.

## Data Availability

The data used in this study is available from the corresponding author, O.A.R. upon reasonable request.

## Appendix A

**Table A1:** Robustness of the density–structure interaction: alternative samples and influential-city checks.

Variables	Main: Full Sample	Main: Drop 2021	Main: Exclude City 2 & 23	Robust: Drop Big Cities
L.ln(LP)	0.453 ***	0.420 ***	0.442 ***	0.418 ***
	(0.0634)	(0.0648)	(0.0626)	(0.0657)
ln(Dens)	1.078 ***	1.339 *	1.064 ***	0.969 **
	(0.276)	(0.786)	(0.291)	(0.471)
ln(Struct)	0.189 ***	0.197 ***	0.222 ***	0.190 ***
	(0.0325)	(0.0339)	(0.0302)	(0.0343)
c.ln(Dens)#c.ln(Struct)	0.0994 **	0.107 ***	0.103 **	0.0976 **
	(0.0380)	(0.0369)	(0.0414)	(0.0424)
Soe_Emp	0.569 ***	0.611 ***	0.646 ***	0.618 ***
	(0.134)	(0.145)	(0.141)	(0.147)
Pri_Emp	−0.836 ***	−0.814 ***	−0.815 ***	−0.780 ***
	(0.260)	(0.270)	(0.264)	(0.282)
Edu	−0.0251	−0.0354	−0.0260	−0.0628
	(0.0381)	(0.0379)	(0.0351)	(0.0436)
Health	−0.223	−0.261	−0.208	−0.467
	(0.274)	(0.300)	(0.314)	(0.307)
2009.year	0.125 ***	0.131 ***	0.130 ***	0.140 ***
	(0.0212)	(0.0216)	(0.0212)	(0.0228)
2010.year	0.107 **	0.122 ***	0.0978 **	0.131 ***
	(0.0422)	(0.0433)	(0.0419)	(0.0471)
2011.year	0.266 ***	0.281 ***	0.254 ***	0.306 ***
	(0.0484)	(0.0512)	(0.0484)	(0.0538)
2012.year	0.337 ***	0.358 ***	0.328 ***	0.386 ***
	(0.0623)	(0.0623)	(0.0625)	(0.0638)
2013.year	0.375 ***	0.403 ***	0.372 ***	0.427 ***
	(0.0724)	(0.0743)	(0.0733)	(0.0778)
2014.year	0.387 ***	0.423 ***	0.384 ***	0.445 ***
	(0.0706)	(0.0723)	(0.0715)	(0.0737)
2015.year	0.397 ***	0.438 ***	0.405 ***	0.452 ***
	(0.0775)	(0.0804)	(0.0783)	(0.0821)
2016.year	0.490 ***	0.530 ***	0.501 ***	0.537 ***
	(0.0810)	(0.0834)	(0.0827)	(0.0840)
2017.year	0.512 ***	0.558 ***	0.531 ***	0.561 ***
	(0.0887)	(0.0933)	(0.0902)	(0.0954)
2018.year	0.586 ***	0.632 ***	0.596 ***	0.624 ***
	(0.0847)	(0.0903)	(0.0862)	(0.0893)
2019.year	0.631 ***	0.681 ***	0.646 ***	0.664 ***
	(0.0878)	(0.0937)	(0.0890)	(0.0938)
2020.year	0.549 ***	0.599 ***	0.557 ***	0.588 ***
	(0.113)	(0.116)	(0.117)	(0.120)
2021.year	0.709 ***		0.723 ***	0.707 ***
	(0.120)		(0.124)	(0.189)
Constant	0.966 ***	0.900	0.984 ***	1.151 ***
	(0.312)	(0.533)	(0.322)	(0.377)
Observations	451	442	424	397
R^2^	0.784	0.778	0.785	0.791
Number of Cities	34	34	32	30

Notes: Standard errors are reported in parentheses on the line below each coefficient. *** *p* < 0.01, ** *p* < 0.05, * *p* < 0.10.

**Table A2:** Robustness of sectoral entropy and alternative samples, and “over-control” specification.

Variables	Entropy: Base Specification	Entropy: Drop 2020-21	Entropy: Exclude City 2 & 23	Entropy: + Struct (Over-Control Check)
L.ln(LP)	0.393 ***	0.388 ***	0.386 ***	0.345 ***
	(0.0673)	(0.0604)	(0.0674)	(0.0632)
ln(Dens)	2.415 ***	1.985 **	2.482 ***	2.583 ***
	(0.794)	(0.845)	(0.843)	(0.815)
H_sector19	1.653 ***	1.565 ***	1.527 ***	1.806 ***
	(0.343)	(0.325)	(0.337)	(0.308)
ln(Dens) × H_sector19	−1.727 ***	−1.483 ***	−1.838 ***	−1.633 ***
	(0.267)	(0.245)	(0.266)	(0.265)
ln(Struct)				0.223 ***
				(0.0400)
Soe_Emp	0.535 ***	0.795 ***	0.610 ***	0.540 ***
	(0.148)	(0.178)	(0.147)	(0.150)
Pri_Emp	−1.156 ***	−0.922 ***	−1.130 ***	−1.093 ***
	(0.226)	(0.201)	(0.229)	(0.219)
Edu	0.0229	0.0306	0.0320	−0.00790
	(0.0390)	(0.0390)	(0.0373)	(0.0394)
Health	−0.189	−0.0586	−0.211	−0.274
	(0.288)	(0.326)	(0.343)	(0.314)
Constant	−0.0142	−0.0317	0.0926	−0.179
	(0.458)	(0.476)	(0.487)	(0.524)
Year FE	Yes	Yes	Yes	Yes
City FE	Yes	Yes	Yes	Yes
Observations	440	407	414	440
R^2^	0.787	0.814	0.783	0.818
Number of Cities	34	34	32	34

Notes: Standard errors are reported in parentheses on the line below each coefficient. Year-dummy coefficients are omitted for brevity. *** *p* < 0.01, ** *p* < 0.05.

**Table A3:** Robustness of ownership entropy and alternative samples, and “over-control” specification.

Variables	Entropy: Base Specification	Entropy: Drop 2020-21	Entropy: Exclude City 2 & 23	Entropy: +SOE & +PRI (Over-Control Check)
L.ln(LP)	0.462 ***	0.476 ***	0.444 ***	0.370 ***
	(0.0751)	(0.0691)	(0.0783)	(0.0636)
ln(Dens)	0.006	0.212	−0.014	0.756
	(0.797)	(0.875)	(0.850)	(0.662)
H_own4	1.075 ***	0.901 **	1.143 ***	1.309 ***
	(0.370)	(0.355)	(0.380)	(0.331)
ln(Dens) × H_own4	0.747 **	0.783 **	0.866 **	0.465 *
	(0.349)	(0.356)	(0.355)	(0.246)
ln(Struct)	0.254 ***	0.242 ***	0.260 ***	0.250 ***
	(0.0374)	(0.0364)	(0.0413)	(0.0370)
Soe_Emp				0.701 ***
				(0.132)
Pri_Emp				0.134
				(0.279)
Edu	−0.0453	−0.0165	−0.0436	−0.0298
	(0.0595)	(0.0546)	(0.0636)	(0.0428)
Health	0.261	0.186	0.259	0.0545
	(0.337)	(0.313)	(0.427)	(0.244)
Constant	0.144	0.124	0.0883	−0.377
	(0.388)	(0.438)	(0.434)	(0.586)
Year FE	Yes	Yes	Yes	Yes
City FE	Yes	Yes	Yes	Yes
Observations	442	408	416	442
R^2^	0.777	0.802	0.775	0.815
Number of Cities	34	34	32	34

Notes: Standard errors are reported in parentheses on the line below each coefficient. Year-dummy coefficients are omitted for brevity. *** *p* < 0.01, ** *p* < 0.05, * *p* < 0.10.

**Table A4:** Dynamic-panel and IV robustness checks for the core interaction model.

Variable	Coefficient
Panel A. System-GMM: Core Interaction Model	
L.ln(LP)	0.512 ***
	(0.138)
ln(Dens)	−0.012
	(0.097)
ln(Struct)	0.249 ***
	(0.084)
ln(Dens) × ln(Struct)	0.165 **
	(0.075)
Soe_Emp	−0.084
	(0.240)
Pri_Emp	−0.061
	(0.415)
Edu	−0.014
	(0.030)
Health	0.798 **
	(0.369)
Constant	1.236 **
	(0.479)
Observations	451
Number of cities	34
Number of instruments	34
AR(1) *p*-value	0.001
AR(2) *p*-value	0.136
Hansen *p*-value	0.482
City FE	Yes
Year FE	Yes
Panel B. Anderson–Hsiao IV: Core Model (First Differences)	
ΔL.ln(LP)	0.577 ***
	(0.108)
Δln(Dens)	1.771 ***
	(0.548)
Δln(Struct)	0.136 ***
	(0.033)
Δ[ln(Dens) × ln(Struct)]	0.155 **
	(0.066)
ΔSoe_Emp	0.546 ***
	(0.183)
ΔPri_Emp	−1.576 ***
	(0.362)
ΔEdu	−0.006
	(0.044)
ΔHealth	−0.111
	(0.241)
Constant	0.412 ***
	(0.145)
Observations	417
Number of cities	34
City FE	Yes
Year FE	Yes

Notes: Panel A reports two-step robust System-GMM estimates with collapsed instruments and lag window 2–3. Panel B reports Anderson–Hsiao IV estimates in first differences, where the differenced lagged dependent variable is instrumented with L2.ln(LP). Standard errors are reported in parentheses on the line below each coefficient. Year-dummy coefficients are omitted for brevity. *** *p* < 0.01, ** *p* < 0.05.

**Table A5:** Multicollinearity diagnostics for the main specifications.

Variable	Main Density–Structure Model	Sectoral Entropy Model	Ownership Entropy Model
L.ln(LP)	2.49	2.71	2.30
ln(Dens)	2.06	1.86	1.75
ln(Struct)	2.27		2.11
Sectoral entropy (H_sector19)		1.77	
Ownership entropy (H_own4)			1.27
Soe_Emp	1.65	1.62	
Pri_Emp	1.85	1.98	
Edu	4.43	4.35	4.24
Health	4.99	4.39	4.68
Mean VIF	2.51	2.49	2.48
Max VIF	4.99	4.39	4.68

Notes: VIFs are computed from auxiliary OLS specifications using the same substantive regressors and year effects as the main models. City fixed effects are excluded from the VIF calculations because a large set of unit dummies can mechanically inflate collinearity diagnostics without being substantively informative. All reported VIF values are below 5.
